# Clinical and Functional Outcomes following Primary Repair versus Reconstruction of the Medial Patellofemoral Ligament for Recurrent Patellar Instability

**DOI:** 10.1155/2014/702358

**Published:** 2014-03-20

**Authors:** Marc Tompkins, Christopher M. Kuenze, David R. Diduch, Mark D. Miller, Matthew D. Milewski, Joseph P. Hart

**Affiliations:** ^1^Department of Orthopaedic Surgery, University of Minnesota, 2450 Riverside Avenue South, Suite R200, Minneapolis, MN 55454, USA; ^2^Department of Orthopaedic Surgery, University of Virginia, Charlottesville, VA 22908, USA; ^3^Elite Sports Medicine, Connecticut Children's Medical Center, Hartford, CT 06032, USA

## Abstract

*Background*. The purpose of this study was to compare outcomes of medial patellofemoral ligament (MPFL) repair or reconstruction. *Methods*. Fourteen knees that underwent MPFL repair and nine (F5, M4) knees that underwent reconstruction at our institution were evaluated for objective and subjective outcomes. The mean age at operation was 20.1 years for repair and 19.8 years for reconstruction. All patients had a minimum of 2 years of follow-up (range: 24–75 months). Patient subjective outcomes were obtained using the International Knee Documentation Committee (IKDC) and Kujala patellofemoral subjective evaluations, as well as Visual Analog (VAS) and Tegner Activity Scales. Bilateral isometric quadriceps strength and vastus medialis obliquus (VMO) and vastus lateralis (VL) surface EMG were measured during maximal isometric quadriceps contractions at 30° and 60° of flexion. *Results*. There were no redislocations in either group. There was no difference in IKDC (*P* = 0.16), Kujala (*P* = 0.43), Tegner (*P* = 0.12), or VAS (*P* = 0.05) scores at follow-up. There were no differences between repair and reconstruction in torque generation of the involved side at 30° (*P* = 0.96) and 60° (*P* = 0.99). In addition, there was no side to side difference in torque generation or surface EMG activation of VL or VMO. *Conclusions*. There were minimal differences found between patients undergoing MPFL repair and MPFL reconstruction for the objective and subjective evaluations in this study.

## 1. Introduction

Patellar instability poses a difficult treatment problem in the setting of recurrent instability [1]. Many types of operative management to address injury to the medial patellofemoral ligament (MPFL) have been described [2]. These include repair of the MPFL, which has been described using arthroscopic, arthroscopically assisted, or open techniques [3–6]. When the pathology is within the midsubstance of the tendon such as chronic stretching of the MPFL, imbrication has been described where the fibers of the MPFL are tightened [7–12]. Finally, when repair and imbrication are not possible or not recommended, reconstruction of the MPFL using various techniques has been described [13–22].

Long term clinical studies evaluating management options for patellar instability have come in many forms. In recurrent patellar instability or chronic injury to the MPFL, repair of the MPFL has provided mixed clinical results [23–34]. Reconstruction of the MPFL in recurrent patellar instability has been evaluated in many long term studies with good results, in general [13, 18, 25–33]. All of the techniques for repair or reconstruction come with their own complications and limitations, and to date the literature is still somewhat unclear about when to employ the various techniques. Potential complications of both MPFL repair and reconstruction include redislocation and postoperative quadriceps dysfunction. MPFL repair has never been compared with MPFL reconstruction in recurrent patellar instability, so it is unclear if one is superior. The purpose of this retrospective study, therefore, was to perform this comparison. Because of the potential for a stronger postoperative construct, the hypothesis was that MPFL reconstruction would provide fewer redislocations, better objective outcomes in terms of quadriceps function, and better subjective outcomes than MPFL repair for the management of recurrent patellar instability.

## 2. Methods

**Figure 1:**
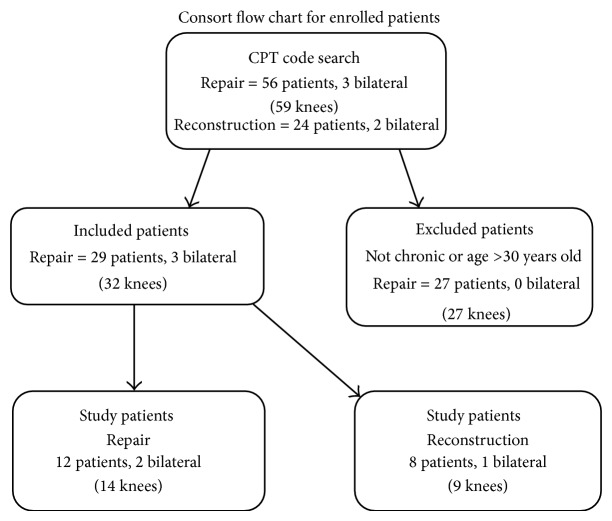
Consort diagram demonstrating patient inclusion and exclusion.

This study was approved by the Institutional Review Board at our institution. The study was initiated by carrying out a broad CPT code search for all MPFL repairs and reconstructions from 2001 to 2009. Each chart retrieved was then reviewed and all patients having a procedure done to the MPFL for recurrent patellar instability were noted. Inclusion criteria were patients undergoing MPFL repair or reconstruction for recurrent patellar instability, defined as two or more instability events. In addition, patients were included based on age from 14 to 30 years. Exclusion criteria were MPFL procedures in acute patellar instability and any procedures that did not include intervention with the MPFL. Patients meeting inclusion criteria were then contacted and invited to participate in the study. Twenty-nine patients met the inclusion criteria for MPFL repair, three of whom were bilateral. Fourteen (44%) knees from twelve patients were included in the study. Eleven patients met the inclusion criteria for MPFL reconstruction, one of whom was bilateral. Nine (75%) knees from eight patients were included in the study. The remainder of the eligible patients either declined to participate or we were unsuccessful in obtaining up-to-date contact information for them and they were lost to follow-up (Figure 1).

Following the chart review, we recorded age at surgery, age at follow-up exam, time to follow-up, gender, BMI, and Insall-Salvati ratio. The Insall-Salvati ratio in all patients was calculated by an orthopedic sports medicine fellowship trained surgeon. All patients returned to participate in the study at a minimum of 2 years following the index procedure, and all follow-ups were carried out within one year of the chart review. At the time of follow up, patients completed the International Knee Documentation Committee (IKDC) subjective knee rating (range 0–100), Kujala patellofemoral score (range 0–100), Tegner Activity Scale (range 0–10), and Visual Analog Scale (VAS) for pain (range 0–10) [34–36].

### 2.1. Strength Testing

**Figure 2:**
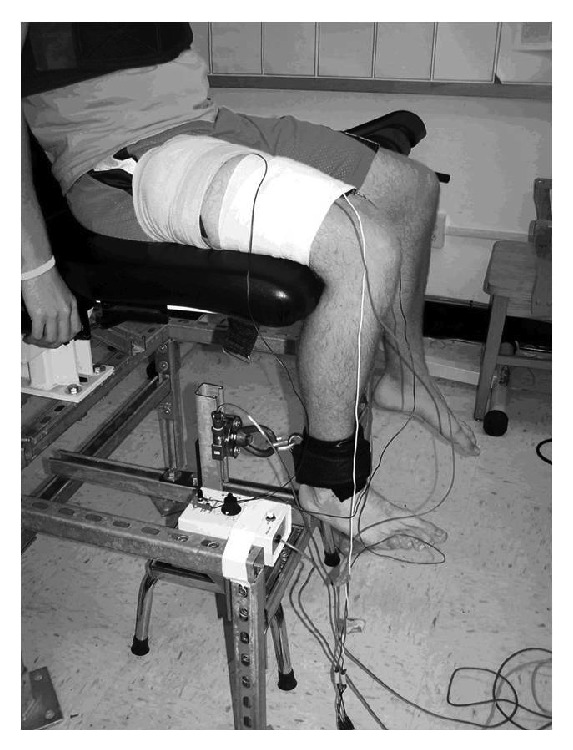
The setup for measuring quadriceps torque generation and surface EMG activation.

All subjects underwent quadriceps strength testing with one consistent researcher experienced with EMG and strength testing. All testing was carried out within one year following the chart review process. We measured bilateral isometric knee extension strength using a multimode dynamometer (Biodex System 3, Shirley, NY). Patient knee, trunk, and hip position were standardized and secured to the chair with straps. The EMG pads were placed over the vastus medialis obliquus (VMO), obliquely oriented just proximal and medial to the patellar base. The vastus lateralis (VL) pad was placed approximately 10 cm proximal to the patellar base and laterally over the muscle belly of the VL, as assessed by palpation during an active knee extension contraction. EMG electrodes were comprised of 2, round, pregelled Ag-AgCl metal discs that were placed on clean, dry skin that was shaved of hair, debrided with a course surface, and cleansed with isopropyl alcohol in order to minimize skin resistance. Electrodes were placed parallel with muscle fiber orientation and at a standard 2 cm interelectrode distance. After multiple practice contractions, each patient performed a 10-second maximal isometric contraction at 60 and 30 degrees of knee flexion (Figure 2). We concurrently recorded VL and VMO surface EMG muscle activation during the maximal tests. Electrodes were placed on shaved, debrided, and cleansed skin, parallel to muscle fiber orientation and approximately 2 cm apart. Signals were amplified, digitized, and processed using a 10-sample moving window root mean square algorithm. The average knee extension torque (normalized to body mass), and corresponding EMG activation from a 5-second time epoch, were calculated for each contraction.

### 2.2. Surgical Intervention

All patients were preoperatively evaluated for pathology of the patellofemoral complex using exam findings, TTTG values on MRI, and trochlear dysplasia findings on plain radiographs and MRI. TTTG values greater than 20, or high Q angles prior to the advent of the TTTG measurement, resulted in anteromedialization (AMZ) osteotomies in addition to proximal realignment procedures. No patients had type C or D trochlear dysplasia and therefore it is not felt that any patient would have benefited from a trochleoplasty [37, 38]. Patients were offered proximal realignment if they had exam findings consistent with MPFL laxity and they had failed a physical therapy program with a focus on patellar stabilization and quadriceps strengthening. Exam findings included lateral patellar translation significantly different from the contralateral side or 3 quadrant translations without an obvious endpoint. MRI findings often, but not always, corroborated the exam findings and involved tearing of the MPFL at the patella, midsubstance, or femur. Patients were selected for MPFL repair if they had clear evidence of MPFL tearing directly from the femur or patella on MRI or if their procedure was performed prior to the routine use of MPFL reconstruction at our institution. Patients not matching these criteria underwent MPFL reconstruction. The two senior surgeons both performed MPFL repairs and reconstructions. All patients underwent arthroscopy at the time of surgery to identify and treat any intra-articular pathology.

During surgery, the isometry of each repair or reconstruction was evaluated. On the femoral side, the position of the repair anchors or femoral tunnel guide pin in reconstructions was checked using intraoperative fluoroscopy; the location on the femur was just anterior (approximately 1 mm) to the extension of the posterior femoral cortex and just distal (approximately 2 mm) to the posterior origin of the medial femoral condyle [39]. In addition, prior to final fixation or femoral tunnel drilling, the repair or autograft was held at the identified femoral insertion while the knee was taken through a range of motion and the MPFL tissue was evaluated for isometry during the range of motion. On the patellar side, the fixation for repair or reconstruction was placed in the anatomic insertion of the MPFL on the patella [40]. After each procedure was completed, all knees were again taken through a range of motion and the patella was felt to appropriately track in all patients.

**Figure 3:**
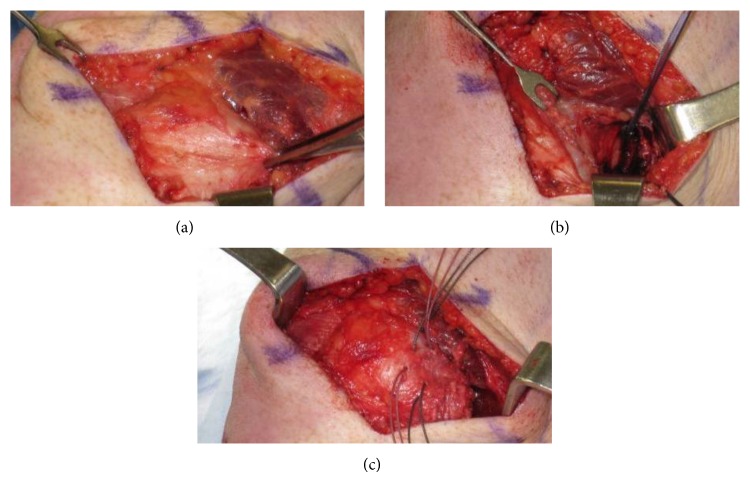
(a) The Allis clamp is being used to evaluate competency of the MPFL after it has been avulsed off the femur. (b) Suture anchors are placed at the MPFL insertion on the femur. (c) The MPFL is sutured back to the femoral insertion using suture anchors.

The repair technique was based on the location of injury along the MPFL. The ligament was identified during dissection; however, often the ligament is difficult to definitively identify so tension was applied to the tissue for confirmation of correct identification. Using suture anchors, the ligament was then secured back to the area of injury and the tissue was tightened (Figure 3). Generally speaking, the location of injury was tearing the ligament off the femur but did sometimes involve the patella. Also included in the repair group were four patients undergoing imbrications in which anchors were not used. Rather, sutures were placed in the midsubstance of the ligament in a “pants-over-vest” fashion in order to tighten the ligament. Imbrication procedures were performed in patients where there was no discrete tear but chronic stretching of the MPFL was obvious on exam; these imbrications were performed prior to reconstructions becoming a routine part of our practice.

**Figure 4:**
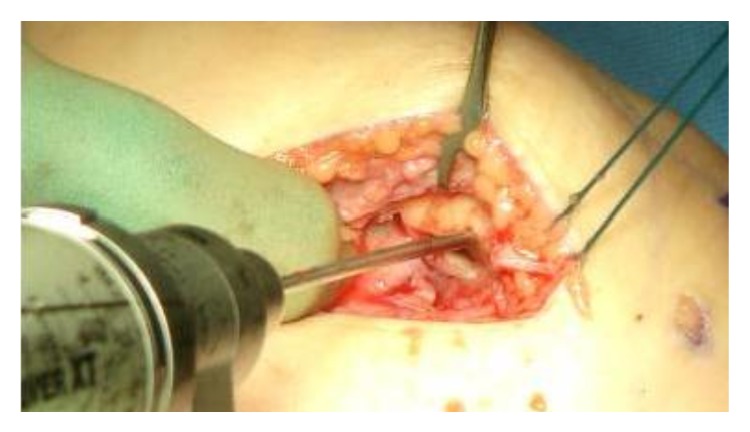
Patellar tunnels are prepared using a drill bit.

The reconstruction technique was completed with the use of hamstring autograft. Once the autograft was harvested, the origin and insertion of the MPFL were identified on the patella and femur. On the patellar side, two drill holes were made through which the ends of the autograft tendon were passed. The ends were then secured either with tenodesis screws or by tying them over one another on the lateral side of the patella. (Figure 4) The autograft tendon was then directed through a drill hole in the femur, tensioned, and secured with a tenodesis screw or fixed with a post and spiked washer (2 patients) on the medial aspect of the femur, at the isometric point [40–42].

### 2.3. Rehabilitation

Patients undergoing either technique were given the same general rehabilitation protocol. All patients were made weight-bearing as tolerated with a hinged knee brace locked in extension for weight-bearing. For the first six weeks postoperatively, patients primarily worked on range of motion exercises with full range of motion allowed as tolerated, when the patient was not weight-bearing. From six weeks to three months, closed chain strengthening was initiated. Open chain strengthening was initiated at three months postoperatively. Finally patients were allowed to return to play at five to six months postoperatively, once they had passed a functional assessment.

### 2.4. Group Comparisons and Subgroup Analysis

Patients undergoing repair and reconstruction were compared using subjective outcomes, strength testing, redislocation rate, Insall-Salvati ratio, length of follow-up, age at surgery and at follow-up exam, and BMI. In addition, two subgroup analyses were performed to compare repair patients with and without concomitant tibial tubercle osteotomies (8 and 6 knees, resp.), as well as to compare reconstructions (9 knees) with repair patients who did not have an osteotomy (6 knees).

### 2.5. Statistical Analysis

Independent samples *t*-tests for comparisons between groups and paired samples *t*-tests were used for side-side comparisons. Mann-Whitney *U* tests or Wilcoxon signed rank sum tests were used if data were not normally distributed. Tests were considered statistically significant if the *P* values were 0.05 or less.

## 3. Results

There were no significant differences in repair versus reconstruction for age at surgery (20.1 versus 19.8; *P* = 0.83), age at follow-up exam (23.5 versus 21.4; *P* = 0.16), and BMI (26.3 versus 23.7; *P* = 0.42). There was a significant difference in average time to follow-up with 43 ± 19.9 months for repair versus 29.2 ± 15.9 months for reconstruction (*P* = 0.05). In the repair group, there were nine females and three males, with two bilateral repairs amongst the females. In the reconstruction group, there were four females and four males, with one bilateral reconstruction amongst the females.

There were no redislocations in either group. One repair experienced a subluxation event but did not require surgery and improved. There were no other complications following these procedures. The Insall-Salvati ratio was not significant between repair (1.1 ± 0.2) and reconstruction (1.3 ± 0.1; *P* = 0.24). Just over half of the repairs (8/14) and none of the reconstructions (0/9) underwent concomitant AMZ osteotomy. One repair undergoing AMZ osteotomy also underwent meniscal repair, and a separate repair patient had a tear in the posterior horn of the lateral meniscus that was not unstable and therefore not repaired. Two repair patients also had loose bodies, which were removed at the time of MPFL repair, but the extent of other concomitant chondral procedures was shaving chondroplasty for partial thickness chondral lesions. Beyond these patients, there were no other concomitant meniscal or cartilage procedures.

There were no statistical differences between repair and reconstruction during strength testing. Specifically, there was no side to side difference between involved and uninvolved sides for normalized isometric knee extension torque generation at 30 degrees or 60 degrees (Table 1). In addition, there was no difference in side to side ratio for surface EMG activation at 30 degrees for VL (*P* = 0.74) or for VMO (*P* = 0.27) (Table 2). There was also no difference in side to side ratio for surface EMG activation at 60 degrees for VL (*P* = 0.46) or for VMO (*P* = 0.73).

The average IKDC score was 75.6 ± 8.0 (range 67.8–87.4) for repair versus 62.3 ± 18.5 (range 40–89) for reconstruction (*P* = 0.16). The average Kujala patellofemoral score was 81.2 ± 8.6 (range 71–98) for repair versus 73.4 ± 19.6 (range 27–92) for reconstruction (*P* = 0.43). The average VAS score was 1.1 ± 0.5 for repair versus 3.1 ± 2.4 for reconstruction (*P* = 0.05). The median preinjury Tegner score was 5.5 (range 3–10) for repair versus 8 (range 6–9) for reconstruction (*P* = 0.03). The median Tegner score at follow-up was 6.5 (range 4–9) for repair versus 5 (range 2–9) for reconstruction (*P* = 0.12). Nine of fourteen repair patients and two of nine reconstruction patients returned to preinjury levels.

In subgroup analysis evaluating repairs with and without AMZ osteotomies, there was no difference in EMG data, IKDC, Kujala, Tegner, and VAS scores. Between repair patients without an AMZ osteotomy and reconstruction patients, there was a difference in VAS scores (1 ± 0 for repair versus 3.1 ± 2.4 for reconstruction; *P* = 0.03) and Tegner score at follow-up (8 (range 5–8) for repair versus 5 (range 2–9) for reconstruction; *P* = 0.02). There was no difference in any other variable between repair patients without an AMZ osteotomy and reconstruction patients.

## 4. Discussion

This study comparing outcomes of repairs and reconstructions for recurrent patellar instability demonstrates minimal difference in outcomes between the two procedures. There were no differences between groups in terms of continued instability. In subjective evaluations, there was less pain on the VAS for repairs, but both groups had fairly low VAS scores. In addition, there was greater return to activity in the repairs; however, the repair group had a lower preinjury Tegner score. In strength and surface EMG testing, there were no significant differences between the two groups.

In MPFL repair for recurrent patellar instability, there are few studies reporting outcomes and they demonstrate varying results. A recent study by Arendt et al. has reported a redislocation rate of nearly 50% for MPFL repair in recurrent patellar instability [23]. Camp et al. found a redislocation rate of 28% [24]. It is unclear why there would be such differences in recurrent instability among those two studies and ours. It is possible, however, that there are important differences in the cohorts of patients. For example, in the case of the study by Arendt et al., there were greater numbers of patients with patella alta and trochlear dysplasia. In the Camp et al. study, half of the redislocation patients had nonanatomic placement of femoral anchors. If these are not included, then the redislocation rate was 16%, which would be closer to our results. When comparing subjective outcomes, the Kujala score in our cohort was slightly lower than that found by Camp et al., but similar postoperative Tegner scores were found.

The literature for MPFL reconstruction in recurrent patellar instability demonstrates lower redislocation rates, anywhere from 0–11% in four recent studies, which is more similar to our cohort [18, 20, 28, 30]. In terms of subjective data, the most commonly reported outcome is the Kujala score. These four studies report Kujala scores of 84, 83, 85.7, and 90.7, which is somewhat higher than the 73.4 found in our reconstruction cohort, although it is unclear why this might be [18, 20, 28, 30]. We were not able to identify studies evaluating the IKDC or VAS scores following MPFL reconstruction; however, one study did report a follow-up Tegner score of 5.1, which is similar to ours [28].

To our knowledge, there are no published studies reporting quadriceps function following MPFL repair or reconstruction. Our data suggests there is no difference in quadriceps strength or EMG quadriceps muscle activation between repair and reconstruction and no side-side differences. Interestingly, the isometric quadriceps strength comparison reported at 60 degrees did not reach statistical significance; however the mean side-side difference was approximately 16%. This may represent either an underlying deficit prior to injury or a potential clinically relevant strength deficit on the operated side in these patients. Either scenario would be similar to prior reports of side-side strength and muscle activation deficits in patients with patellofemoral pain syndrome [43–47]. Our data represents the first time quadriceps function that has been evaluated following MPFL repair or reconstruction and it suggests symmetry in quadriceps function; however, there is a possibility that clinically relevant strength deficits exist.

Given the data from this study, we feel more confident in the viability of repairs for recurrent instability at short to midterm follow-up if the repair is performed in the appropriate patient. Our current protocol for managing recurrent instability is to perform a repair in the setting of an MRI with an obvious MPFL avulsion lesion of the femur or patella. This is then further confirmed during surgery. To identify tears of the femur, the MPFL is localized and held with forceps against the anticipated repair site as manual pressure is applied against the patella laterally. The MPFL is in continuity, and potentially reparable, if holding the tissue at the origin clearly stabilizes the patella once tension is applied. Alternatively, peel-off lesions from the patella can sometimes be visualized arthroscopically. In the setting of a lesion of the patella, holding the MPFL with forceps does not restrict lateral patellar translation, but the surgeon can confirm that the MPFL is anchored at its femoral origin through applied tension. If it is not clear that the ligament has avulsed from the femur or patella, or if there is any concern about the integrity of the ligament, a reconstruction with hamstring autograft is performed.

During the evolution of this protocol, some repairs were performed where no suture anchors were used. Instead, only sutures were used to imbricate the ligament tissue. In our study, these few patients achieved similar subjective and objective outcomes to the remainder of the repair group. This technique, however, is generally no longer performed at our institution because of the potential for a misdiagnosed tear at either the origin or insertion. Instead, we now favor a reconstruction of the MPFL rather than an imbrication in this setting.

It should be noted that a limitation of this study is that there was a difference in the number of AMZ osteotomies performed between groups, which could represent a confounding variable. Based on exam and MRI findings, it was not felt that distal realignment was necessary in any of the reconstruction patients. It is possible, however, that the difference in VAS scores between the total repair group and the reconstruction group could be explained by the fact that AMZ osteotomies have been shown to independently improve pain [48–50]. Interestingly though, the VAS remained lower in the repair group when repairs without osteotomies were compared to the reconstruction group, suggesting that the osteotomies did not contribute to somewhat better scores of the total repair group versus the reconstruction group. In addition, though the numbers within each subgroup are small, there was no difference in EMG data, IKDC, Kujala, Tegner, and VAS scores between repair patients with and without AMZ osteotomy. We, therefore, feel that it is acceptable to compare the total repair group and the reconstruction group directly despite the difference in patients undergoing AMZ osteotomies.

The small sample size, compounded by the number of patients lost to follow-up, is also another limitation of this study. Other recent studies, however, evaluating outcomes in MPFL repair or reconstruction demonstrate that these are not high volume surgeries and therefore large numbers of study patients are difficult to obtain [18, 20, 23, 24, 28, 30]. Another limitation of the study is that the management of recurrent patellofemoral instability is still very much in evolution, and therefore over the course of this study there was some variety in technique used for each procedure. For example, imbrications were initially used as a repair technique and a post and washer were used on the femur for two reconstruction patients. As far as other concomitant procedures, however, there were limited cartilage or meniscus procedures which would potentially affect the data, and no trochleoplasties were performed [51].

Further long term, prospective studies comparing MPFL repair and reconstruction are warranted to help elucidate the best management of recurrent patellar instability and which patients should receive which procedure.

## 5. Conclusion

There were minimal differences found between patients undergoing MPFL repair and MPFL reconstruction for the objective and subjective evaluations in this study.

## Figures and Tables

**Table 1:** Repair versus reconstruction comparison for torque (Nm/kg) generation of involved and uninvolved sides at 30 and 60 degrees of knee flexion.

Flexion angle and side	Repair	95% Confidence intervals	Reconstruction	95% Confidence intervals	*P* value
Torque at 30 degrees, involved	1.09	0.77–1.41	1.07	0.82–1.32	*P* = 0.96
Torque at 30 degrees, uninvolved	1.18	0.91–1.45	1.16	0.92–1.4	*P* = 0.92
Torque at 60 degrees, involved	1.81	1.28–2.34	1.82	1.51–2.13	*P* = 0.99
Torque at 60 degrees, uninvolved	2.17	1.71–2.63	1.91	1.43–2.39	*P* = 0.56

**Table 2:** Side to side comparison for torque (Nm/kg) generation at 30 and 60 degrees of knee flexion.

Flexion angle and side	Involved	95% Confidence intervals	Uninvolved	95% Confidence intervals	*P* value
Torque at 30 degrees	1.09	0.87–1.31	1.17	0.98–1.36	*P* = 0.45
Torque at 60 degrees	1.75	1.4–2.1	2.09	1.77–2.41	*P* = 0.08
